# FAM64A is an androgen receptor-regulated feedback tumor promoter in prostate cancer

**DOI:** 10.1038/s41419-021-03933-z

**Published:** 2021-07-02

**Authors:** Yingchen Zhou, Longhua Ou, Jinming Xu, Haichao Yuan, Junhua Luo, Bentao Shi, Xianxin Li, Shangqi Yang, Yan Wang

**Affiliations:** 1 Department of Urology, Peking University Shenzhen Hospital, Institute of Urology, Shenzhen PKU-HKUST Medical Center, Shenzhen, China; 2 Department of Surgery, Fuwai Hospital Chinese Academic of Medical Science Shenzhen, University of South China, Shenzhen, China; 3 Department of Urology, Shenzhen Second People’s Hospital, The First Affiliated Hospital of Shenzhen University, Shenzhen, China; 4 Department of Urology, Taikang Qianhai International Hospital, Shenzhen, China

**Keywords:** Penile cancer, Predictive markers

## Abstract

Endocrine therapy for prostate cancer (PCa) mainly inhibits androgen receptor (AR) signaling, due to increased androgen synthesis and AR changes, PCa evolved into castration-resistant prostate cancer (CRPC). The function of Family With Sequence Similarity 64 Member A (FAM64A) and its association with prostate cancer has not been reported. In our research, we first reported that FAM64A is up-regulated and positively associated with poor prognosis of patients with prostate cancer (PCa) by TCGA database and immunohistochemistry staining. Moreover, knockdown of FAM64A significantly suppressed the proliferation, migration, invasion, and cell cycle of PCa cells in vitro. Mechanistically, FAM64A expression was increased by dihydrotestosterone (DHT) through direct binding of AR to FAM64A promoter, and notably promoted the proliferation, migration, invasion, and cell cycle of androgen-dependent cell line of PCa. In addition, abnormal expression of FAM64A affects the immune and interferon signaling pathway of PCa cells. In conclusion, FAM64A was up-regulated by AR through directly binding to its specific promoter region to promote the development of PCa, and was associated with the immune mechanism and interferon signaling pathway, which provided a better understanding and a new potential for treating PCa.

## Introduction

Prostate cancer (PCa) is one of the most common malignant tumors in the male urinary tract and its incidence ranks fifth among malignant tumors worldwide and second among male malignancies. According to the statistics, the total number of global cases of PCa in 2016 is 1.4 million, and the total number of deaths is 375,304 [1]. Due to PCa patients have no obvious symptoms in the early stage, the majority of them are diagnosed in the late stage, resulting in a low cure rate and higher mortality rate. For patients with advanced PCa, the current standard first-line treatment is androgen deprivation treatment (ADT), which can effectively reduce tumor burden, improve patient quality of life and prolong overall survival time. However, after ADT treatment with a median time of 18–24 months, almost all patients gradually evolved into castration-resistant prostate cancer (CRPC) [2], and 86% of CRPC eventually turned into metastatic castration-resistant prostate cancer (mCRPC) [3]. Interestingly, activated AR molecules can both enhance and inhibit the expression of genes related to PCa progression. This hormone-driven AR signal is essential for the development, differentiation, and normal function of the prostate. Correspondingly, CRPC continues to express AR and AR regulatory genes after androgen ablation therapy [4].

The human FAM64A gene (Family With Sequence Similarity 64 Member A, also known as RCS1, PIMREG, or CATS) is located on chromosome 17p13 and contains eight exons. The mRNA of human FAM64A is about 1517 bp (NM_001195228.1), which encodes a 248 amino acid protein (NP_001182157.1) [5]. FAM64A plays important biological functions in various cells by accelerating the cell cycle. Initial research found that the mutations of the FAM64A gene could regulate the size of *Saccharomyces cerevisiae* cells [6]. FAM64A gene was highly expressed in cardiomyocytes of mouse embryonic period, and it was proved that FAM64A could participate in the process of the cell cycle as a regulator of mitosis [7]. The expression of the FAM64A gene is abnormally regulated in a variety of tumor tissues. Current studies have found that FAM64A was remarkably highly expressed in tumor tissues and cells of patients with leukemia [7], breast cancer [8], and pancreatic cancer [9], which may be related to cell cycle disorders, but its specific molecular mechanism is still unclear.

Janus kinase signal transducer and activator of transcription (JAK–STAT) signal transduction were found in almost all immunoregulatory processes, including those involved in tumor cell recognition and tumor-driven immune escape. STAT activation of countless cytokines and growth factors induces driving events such as hematopoiesis, immune adaptation, inflammation, tissue repair, adipogenesis, and apoptosis. Most immune responses triggered by cytokines rely on STATs. This pathway was first discovered in the framework of interferon (IFN)-signaling research. The anti-tumor immune response is largely driven by STAT1 and STAT2 induced type I and II IFN and downstream program-enhanced IFN [10].

In the present study, we found that FAM64A was overexpressed in PCa tissues and cells, and its expression was positively correlated with poor prognosis in PCa patients. FAM64A expression was activated by AR through direct binding of AR to FAM64A promoter, and notably promoted the proliferation, migration, invasion, and cell cycle of PCa cells. In addition, abnormal expression of FAM64A affected the immune mechanism and IFN signaling pathway in PCa. The results indicate that the DHT/AR–FAM64A–IFN axis may serve as a novel promising diagnostic and therapeutic target for PCa.

## Results

### FAM64A is upregulated in PCa tissues and cell lines

**Fig. 1:**
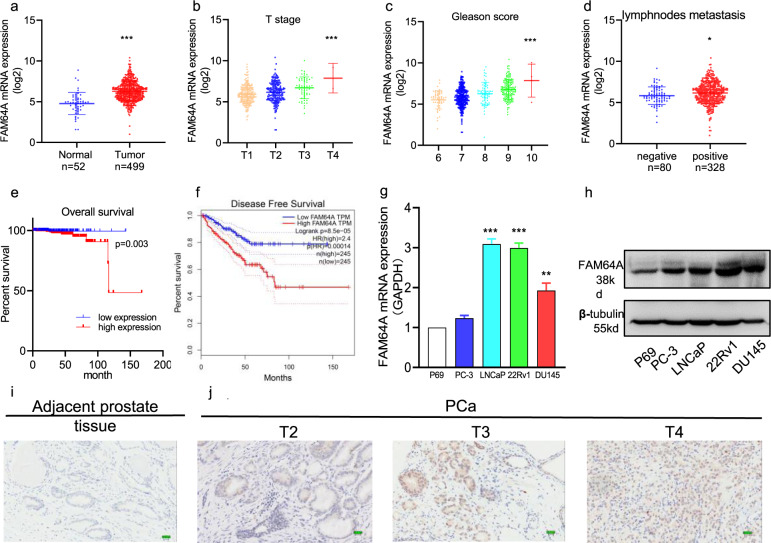
FAM64A is upregulated in prostate cancer tissues and cell lines. **a** The expression of FAM64A in PCa tissues was significantly higher than that in normal prostate tissues by TCGA. **b**–**d** The expression of FAM64 mRNA was related to the T grade, Gleason score, and lymph nodes metastasis. **e**, **f** The lower expression level of FAM64A led to longer overall survival time and disease-free survival time. **g**, **h** FAM64A was remarkably increased in PCa cell lines PC-3, LNCaP, 22Rv1, and DU145 compared to P69 at both transcription and translation levels. **i, j** The expression of FAM64A protein in PCa was higher than that in adjacent normal PCa tissues and was related to the Tumor stage by immunohistochemistry staining. Scale bar = 20.25 μm. *P < 0.05, **P < 0.01, ***P < 0.001.

To investigate the role of FAM64A in the progress of PCa, we first analyzed the expression level of FAM64A in 499 cases of PCa tissue and 52 cases of normal prostate tissue from the TCGA database. It was shown that the FAM64A mRNA level was significantly higher in PCa tissue compared with normal prostate tissue (Fig. 1a). Furthermore, we analyzed the correlation of FAM64A expression and clinicopathological characteristics of 499 patients with PCa. The dysregulation of FAM64A in PCa was associated with the clinical tumor stage, lymph node metastasis, and Gleason score, but not the age of patients with PCa (Fig. 1b–d and Table 2). In addition, the Kaplan–Meier survival analysis and the Kaplan–Meier disease-free survival analysis showed a poor prognosis for PCa patients displaying high expression levels of FAM64A (Fig. 1e, f).

**Table 1:** Expression of FAM64A in PCa and adjacent normal prostate tissues.

FAM64A	No. of cases	Type of tissues		*χ*^2^	*P*
		Negative	Positive		
Adjacent tissue	47(32.6%)	14(9.7%)	33(22.9%)	14.751	0.0001*
PCa	97(67.4%)	6(4.2%)	91(63.2%)		

Pearson’s *X*^2^ test.

***P values < 0.001.

**Table 2:** Correlation between FAM64A expression and the clinicopathological characters of patients with prostate cancer (TCGA).

Clinico-pathologic variables	No. of cases	FAM64a expression		*X*^2^	*P*
		Low	High		
All cases	499	249 (44.3%)	250 (55.7%)		
*Age*					
≤60	224 (44.9%)	122 (24.5%)	102 (20.4%)	3.3871	0.0657
>60	275 (45.1%)	127 (25.4%)	148 (29.7%)		
*Clinical stage*					
T1–T2	350 (86.6%)	188 (46.5%)	162 (40.1%)	10.861	0.0010***
T3–T4	54 (13.4%)	16 (4.0%)	38 (9.4%)		
*Lymph nodes status*					
Negative	328 (80.4%)	161 (39.5%)	167 (40.9%)	5.1321	0.0235*
Positive	80 (19.6%)	28 (6.9%)	52 (12.7%)		
*Gleason score*					
6–7	295 (59.1%)	176 (35.3%)	119 (23.8%)	27.501	<0.0001***
8–10	204 (40.9%)	73 (14.6%)	131 (26.3%)		
*PSA*					
−	429 (96.6%)	213 (48.0%)	216 (48.6%)	0.6210	0.4307
+	15 (3.4%)	9 (2.0%)	6 (1.4%)		
*Survival times (years)*					
≤5	414 (83%)	217 (43.5%)	197 (39.5%)	6.1521	0.0131*
>5	85 (17%)	32 (6.4%)	53 (10.6%)		

Pearson’s *X*^2^ test.

*P values < 0.05, ***P values < 0.001.

The above results were also identified by the IHC data. There was a significant increase of FAM64A protein in 97 cases of PCa tissues compared to the adjacent normal prostate tissues (Fig. 1i, j, Table 1). In addition, the expression of FAM64A was related to the patient’s Lymph node status, Ki-67, TOPO-II, ERG, and the difference was statistically significant (*P* < 0.05) (Table 3). The multivariate Cox-regression analysis showed that the patient’s T stage was related to survival time, and the risk coefficients were 0.001 (Table 4). In summary, our results indicated that FAM64A expression was up-regulated in PCa tissues and cells, and was associated with PCa progression.

**Table 3:** Correlation between FAM64A expression and the clinicopathological characters of patients with PCa (IHC).

Clinico-pathologic variables	No. of cases	FAM64A expression		*χ*^2^	*Ρ*
		low	high		
All cases	97	43(44.3%)	54(55.7%)		
*Age*					
≤60	4(4.1%)	1(1.0%)	3(3.1%)	0.66121	0.4162
>60	93(95.9%)	42(43.3%)	51(52.6%)		
*Tumor stage*					
T1–T2	14(15.4%)	5(5.5%)	9(9.9%)	0.45621	0.4994
T3–T4	77(84.6%)	35(38.5%)	42(46.1%)		
*Lymph nodes status*					
Negative	72(79.1%)	37(40.6%)	35(38.5%)	5.5891	0.0181*
Positive	19(20.9%)	4(4.4%)	15(16.5%)		
*Gleason score*					
6–7	51(55.6%)	21(21.6%)	30(34.0%)	0.43331	0.5104
8–10	46(47.4%)	22(22.7%)	24(24.7%)		
*Ki-67*					
<10	74(79.6%)	39(42.0%)	35(37.6%)	6.0921	0.0136*
>10	19(20.4%)	4(4.3%)	15(16.1%)		
*P53*					
+	21(23.9%)	7(8.0%)	14(15.9%)	1.3491	0.2455
−	67(76.1%)	32(36.3%)	35(39.8%)		
*PSA*					
+	81(85.3%)	38(40.0%)	43(45.3%)	0.079001	0.7787
−	14(14.7%)	6(6.3%)	8(8.4%)		
*SMA*					
+	11(13.6%)	5(6.2%)	6(7.4%)	0.0052601	0.9422
−	70(86.4%)	31(38.3%)	39(48.1%)		
*TOPO-II*					
+	65(81.25%)	23(28.75%)	42(54.5%)	9.8581	0.0017**
−	15(18.75%)	12(15%)	3(3.75%)		
*ERG*					
+	20(25.6%)	3(3.8%)	17(21.8%)	8.9411	0.0028**
−	58(74.4%)	31(39.8%)	27(34.6%)		

Pearson^,^s *X*^2^ test.

*P values < 0.05, **P values < 0.01.

**Table 4:** Multivariate Cox-regression analysis for patients after surgery (TCGA).

Variate	*P*	Exp(B)	95.0% CI	
			Low limit	Up limit
Age	0.882	1.316	0.035	49.998
T stage	0.015*	0.001	0.000	0.239
Gleason score	0.749	0.514	0.009	30.408
Lymph nodes status	0.771	2.040	0.017	248.528
FAM64A	0.937	1.144	0.040	33.061
PSA	0.999	1.032	0.000	8.656E + 17

Cox regression analysis.

*P values < 0.05.

For further study, FAM64A expression levels at both transcriptional and translation levels were detected by qPCR and Western blotting, respectively. It was revealed that FAM64A was overexpressed in PCa cells (PC-3, LNCaP, 22Rv1, and DU145) compared to the human prostatic epithelial cells (P69) (Fig. 1g, h). LNCaP and 22Rv1 cell lines were selected for FAM64A knockdown in subsequent experiments.

### FAM64A promotes the proliferation, migration/invasion, and cell cycle of PCa cells

**Fig. 2:**
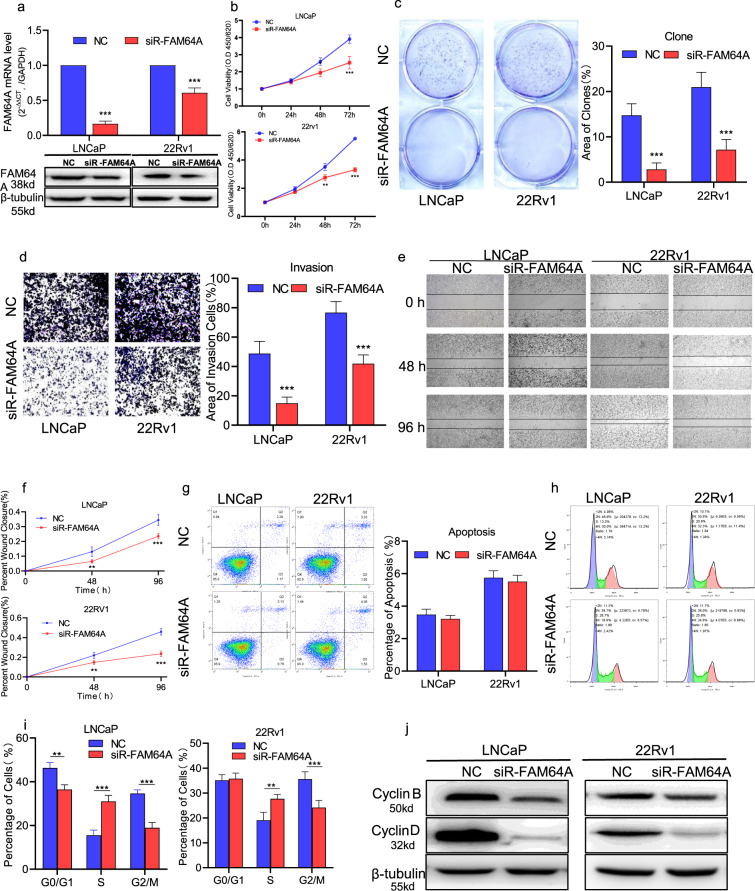
FAM64A promotes the proliferation, migration/invasion, and cell cycle of PCa cells. **a** The mRNA and protein level of FAM64A in PCa cell lines LNCaP and 22Rv1 with transfection of siRNA-FAM64A was noteworthy lower than that in the control group. **b**, **c** CCK-8 assay and colony formation assay showed that knockdown of FAM64A decreased the proliferation of PCa cells. **d** Transwell invasion assay indicated that knockdown of FAM64A reduced the invasive activity of PCa cells. **e**, **f** Wound healing assay indicated that knockdown of FAM64A reduced the migratory ability of PCa cells at 0, 48, 96 h. **g** Flow cytometry with Annexin V-FITC and PI staining indicated that knockdown of FAM64A could not impact cell apoptosis. **h**, **i** Flow cytometry analysis indicated that knockdown of FAM64A postponed the progression of the cell cycle. **j** Western blot analysis showed that knockdown of FAM64A downgulated the expression of cycle-related protein cyclin B1 and cyclin D. **P < 0.01, ***P < 0.001.

To explore the biological function of FAM64A in PCa cells, we transfected FAM64A-specific siRNAs or negative control siRNAs in LNCaP and 22Rv1 cells and analyzed the efficacy of siRNAs knocking down FAM64A. The qPCR and Western blotting results demonstrated that FAM64A expression was decreased in both mRNA and protein levels (Fig. 2a). Further, we detected the role of FAM64A on cell proliferation, migration/ invasion, and apoptosis of PCa cells by CCK-8, wound-healing/transwell-invasion assays, and flow cytometric analysis, respectively. The CCK-8 results showed that the knockdown of FAM64A dramatically inhibited the proliferation of LNCaP and 22Rv1 cells (Fig. 2b). Colony formation data revealed that the growth of LNCaP and 22Rv1 cells was significantly inhibited by siR-FAM64A (Fig. 2c). The Transwell and wound-healing analysis indicated that siR-FAM64A reduced the invasion and migration abilities of LNCaP and 22Rv1 cells strikingly (Fig. 2d–f). The silence of FAM64A had no effect on the apoptosis of PCa cells (Fig. 2g). As shown in Fig. 2h and i, siR-FAM64A cells comprised a significantly lower proportion of cells in the G2-M phase compared to the control. In addition, the protein analysis showed that cycle-related protein cyclin B1 and cyclin D were positively correlated with FAM64A protein expression (Fig. 2j). These results suggested that FAM64A plays a role in promoting tumorigenesis of PCa.

### DHT activated the AR to regulate the expression of FAM64A by targeting its promoter sequences

**Fig. 3:**
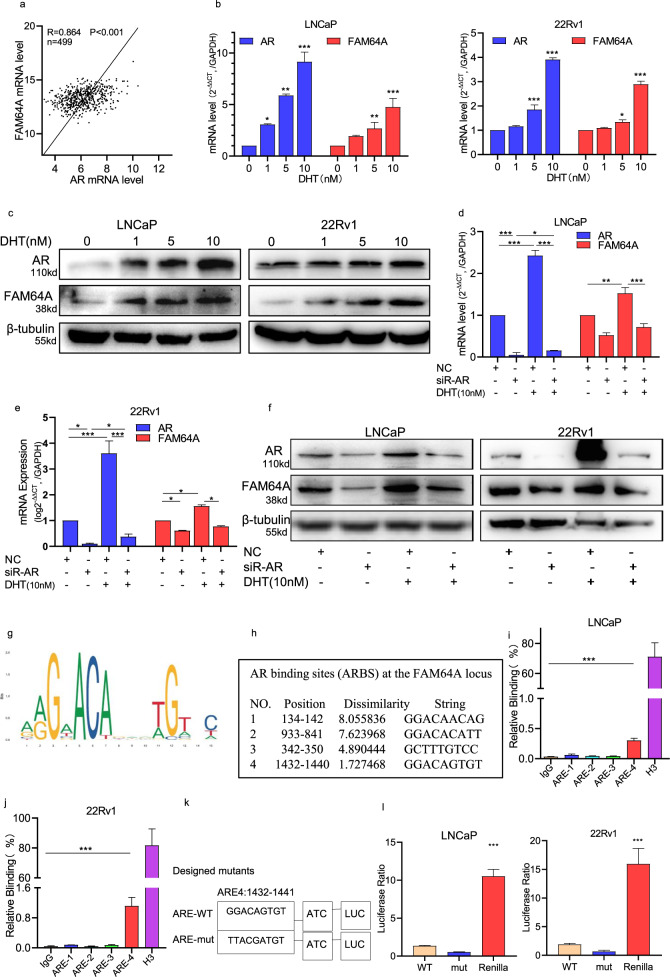
DHT activated the AR to regulate the expression of FAM64A by targeting its promoter sequences. **a** The linear correlation analysis was a positive correlation between AR expression and FAM64A expression in prostate cancer patients by TCGA database. **b**, **c** The expression of FAM64A was accompanied by increased DHT (10 nM) induced AR at both transcription and translation levels. **d**–**f** The mRNA expression of AR and FAM64A in cells transfected with siRNA-AR and DHT (10 nM) at both transcription and translation levels. **g** Canonical full‐ARE sequences. **h** The AR‐binding sites in FAM64A promoter from ChIP‐seq results and the prediction by website JASPAR. **i**, **j** DNA immunoprecipitated with AR antibody and IgG (as a nonspecific control) were used as templates for PCR using primers that amplified the AR-binding sites within the FAM64A promoter. **k** The designed mutants of key residues in the ARE4 *T*-test. **l** LNCaP and 22Rv1 cells were cotransfected with reporter plasmids (containing the wide type and mutants of ARE), Renilla luciferase reporter gene, and AR expression plasmid and then harvested to measure luciferase activity treat the data as above. *P < 0.05, **P < 0.01, ***P < 0.001.

To determine if FAM64A is an androgen-regulated gene, we preliminarily searched the NURSA database and found that the FAM64A mRNA level was regulated after treatment of androgen in PCa cells (Table 5). Furthermore, Pearson’s correlation analysis revealed that the expression of FAM64A was positively correlated with the expression of AR in prostate cancer (TCGA: *P* < 0.001, *R* = 0.864, Fig. 3a). To verify the above result, PCa cells cultured with hormone-free CSS medium were treated with different concentrations of DHT. Then the FAM64A or AR expression was examined by qRT-PCR and Western blotting. In the AR-positive human PCa cell lines LNCaP and 22Rv1, FAM64A and AR were elevated in a dose-dependent manner by low concentration of androgen at both the mRNA (Fig. 3b) and protein level (Fig. 3c). Knockdown of AR attenuated the effect of DHT on FAM64A mRNA (Fig. 3d, e) and protein expression (Fig. 3f), confirming the requirement of AR for DHT-mediated promotion of FAM64A.

**Table 5:** The effect of different concentrations of DHT on the expression of FAM64A.

Group	KD/NC	DHT/Veh (100 nM)	DHT/Veh (10 nM)	ELK1 + R1881/Veh
FAM64A fold change	−3.035	−2.36	3.45	2.38
*P* score	0.000229***	0.00352**	0.00003***	0.00242**

*t*-test analysis.

**P values < 0.01, ***P values < 0.001.

To identify that FAM64A is a direct target gene of AR, we performed the JASPAR database to predict the androgen response sequence in the promoter region of the FAM64A gene (Fig. 3g). There were four potential androgen response sequences in the promoter region of the FAM64A gene (Fig. 3h). Further, the analysis of ChIP-qPCR data revealed a putative AR-binding site within the promoter region of the FAM64A gene following androgen treatment (Fig. 3i, j). To confirm that these binding sites were responsible for AR regulation, we cloned the FAM64A wild or mutant promoter upstream of luciferase and conducted reporter assays (Fig. 3k, l). As expected, the transcriptional activity of this construct was promoted by androgens in LNCaP and 22Rv1 cells. Together, these data indicate the direct transcriptional control of AR on FAM64A gene.

### AR-mediated FAM64A expression regulates cell proliferation, migration, invasion, and cell cycle of PCa cells

**Fig. 4:**
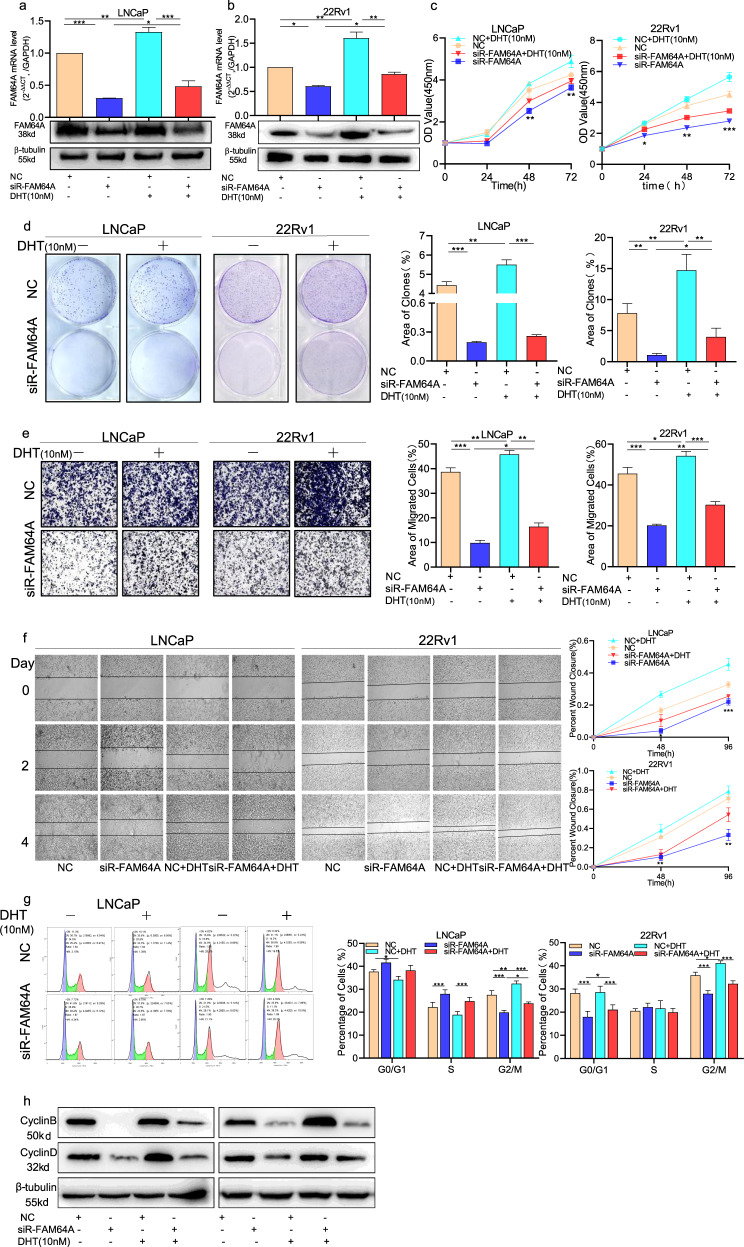
AR-mediated FAM64A expression regulates cell proliferation, migration, invasion, and cell cycle of PCa cells. **a**, **b** The mRNA expression of FAM64A in cells transfected with siR-FAM64A and DHT (10 nM) at both transcription and translation levels. **c**, **d** CCK-8 assay and colony formation assay showed that DHT inhibits the effect of knocking out FAM64A on androgen-dependent PCa cell proliferation. **e** Transwell invasion assay indicated that DHT suppresses the effect of knocking out FAM64A about androgen-dependent PCa cell invasion. **f** Wound healing assay indicated that DHT restrains the effect of knocking out FAM64A about androgen-dependent PCa cell migration. **g** Flow cytometry analysis indicated that DHT moderates the effect of knocking out FAM64A on the progression of the androgen-dependent PCa cell cycle. **h** Western blot analysis showed that the FAM64A expression was upregulated after transfected DHT in siR-FAM64A or control siRNA PCa cells. *P < 0.05, **P < 0.01, ***P < 0.001.

To assess the functional significance of AR-mediated FAM64A expression in PCa cells, PCa cell lines LNCaP and 22Rv1 were treated with DHT and/or transfected by FAM64A-specific siRNA. The qPCR results showed that the expression of FAM64A decreased by siR-FAM64A in PCa cells was reversed after the DHT treatment (Fig. 4a, b). Further, the knockdown of FAM64A inhibits cell proliferation of PCa cells. Interestingly, exposure to DHT enhanced FAM64A-mediated PCa cell proliferation (Fig. 4c). At the same time, clonogenic analysis confirmed that DHT promoted the colony formation capability of FAM64A-mediated PCa cells (Fig. 4d). In addition, wound healing and transwell invasion analysis revealed that DHT could reverse the inhibition of knocking down FAM64A on the migration and invasion of PCa cells (Fig. 4e, f). As a chromosomal segregating gene, FAM64A was previously confirmed to promote the cell cycle from G2 to M phase. Here exposure to DHT could accelerate the process of FAM64A-mediated cell cycle G2 to M (Fig. 4g) and the expression of cyclin B1 and cyclin D (Fig. 4h). These results suggest that DHT activated AR to affect PCa cell proliferation, migration, invasion, and cell cycle by upregulating FAM64A expression.

### FAM64A inhibits immunity and IFN-signaling pathway in PCa

**Fig. 5:**
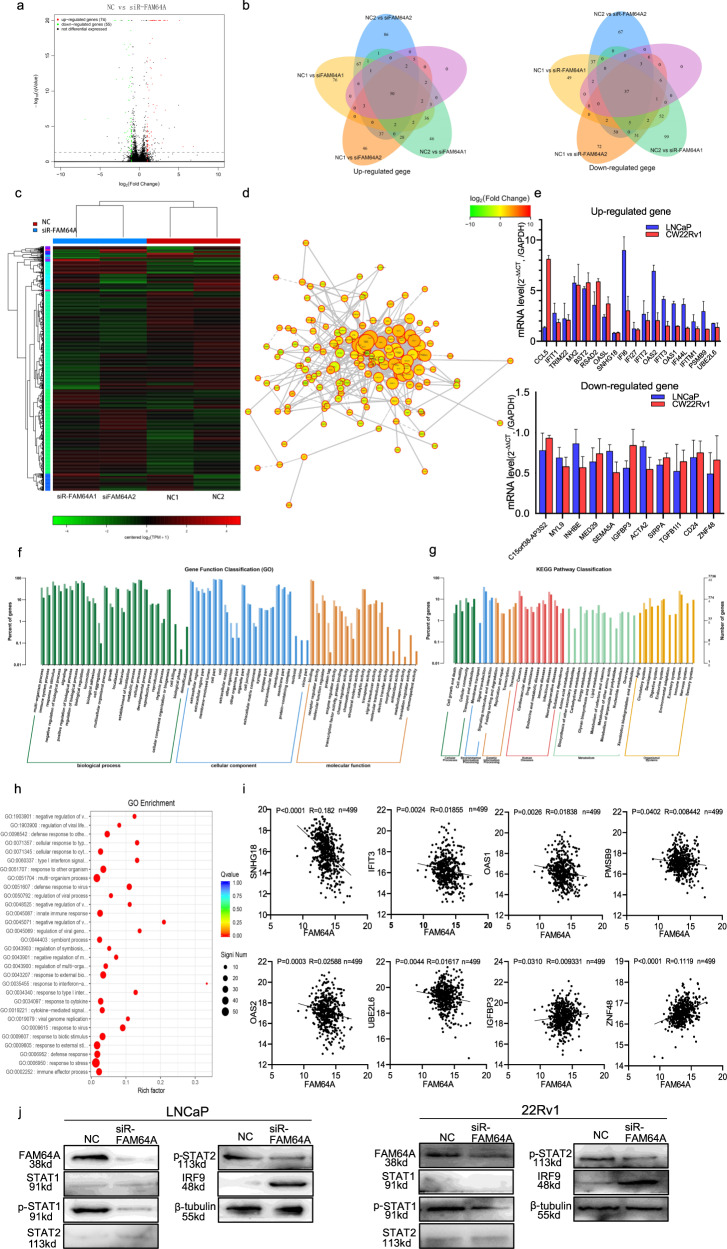
The expression level of FAM64A could regulate the relevant genes of immunity and interferon by the JAK–STAT-signaling pathway in PCa. **a** Red dots represent genes with fold change ≥ 2 and *P* < 0.05, green dots represent genes with fold change ≤ −2 and *P* < 0.05, and the other dots represent the rest of genes with no statistically significant change in NC and siRNA-FAM64A cells by Volcano map. FC fold change. **b** Venn diagram of up-regulated genes and down-regulated genes. **c** The red color represents up-regulated genes and the green color represents down-regulated genes in Heat maps. **d** Construction of protein–protein interaction (PPI) network of differential expressed genes (DEGs), Red circles are upregulated genes in the PPI network, while green ones are downregulated. **e** The q-PCR experiment verifies that the highest log2FoldChange value of 18 up-regulated genes and 13 down-regulated genes which had been found by transcriptome sequencing to siRNA-NC and siRNA-FAM64A PCa cell lines. **f**, **g** The biological functions of the integrated DEGs were explored by GO and KEGG enrichment analysis. **h** The upregulated genes and downregulated genes were subjected to the DAVID for functional pathway analysis by GO enrichment. **i** By the TCGA database, the linear relationship of the seq-HTSeq-FPKM-UQ value of FAM64A in prostate cancer patients was analyzed. It was found that the expression level of SNHG18, IFIT3, OAS1, PMSB9, OAS2, UBE2L6, IGFBP3, ZNF48 was linearly related to the expression level of FAM64A, consistent with sequencing results. **j** Western blot results of key proteins expression for IFN and JAK–STAT-signaling pathway upon the knockdown of FAM64A.

To investigate the molecular mechanisms of FAM64A in process of PCa, we introduced transcriptome sequencing. We applied the GEO2R to identify differential expression analysis sequence (DEGs) regulated by FAM64A in PCa cells. 129 DEGs (74 upregulated genes and 55 downregulated genes) were identified in the siR-FAM64A cell samples and siR-NC samples. We set the cut-off criterion as *P* < 0.05 and [logFC] ≥ 2 (Fig. 5a). The Venn software was used to screen 50 overlapping up-regulated genes and 37 overlapping down-regulated genes (Fig. 5b, Table 6). With the aid of the RRA software package, the 87 DEGs in the integrated microarray analysis were located on the heat map (Fig. 5c). Then, we mapped the differentially expressed genes related to FAM64A to the protein interaction network through the STRING database. Key genes analysis was performed according to the topological properties of the genes in the network (Fig. 5d). The qPCR data verified the top 18 up-regulated genes and top 11 down-regulated genes in transcriptome sequencing of siR-NC and siR-FAM64A group PCa cells (Fig. 5e and Table 6).

**Table 6:** Comparison group expression difference analysis result.

Gene name	Mean TPM (B)	Mean TPM (A)	log2 Fold change	*P* value	*q* Value	Result
CCL5	26.0165	2.1782	3.5782	1.59E−39	1.67E−36	Up
IFIT1	96.9303	9.5637	3.3413	2.99E−74	1.56E−70	Up
TRIM22	7.4270	0.7544	3.2994	3.32E−10	4.97E−08	Up
MX2	5.9272	0.6102	3.2801	7.99E−18	2.41E−15	Up
BST2	50.1506	5.5246	3.1823	8.59E−48	1.50E−44	Up
RSAD2	10.3077	1.1719	3.1367	2.30E−43	2.78E−40	Up
OASL	15.5712	1.8245	3.0933	1.27E−40	1.42E−37	Up
IFI6	199.2223	26.6136	2.9041	4.23E−05	0.00219751	Up
IFI27	38.8152	5.7712	2.7497	2.29E−15	5.61E−13	Up
IFIT2	33.3351	5.2074	2.6784	5.72E−34	5.28E−31	Up
OAS2	12.2688	1.9909	2.6235	1.28E−29	8.75E−27	Up
IFIT3	39.7996	6.6145	2.5890	4.83E−58	1.52E−54	Up
OAS1	32.7115	5.8008	2.4955	1.76E−29	1.10E−26	Up
IFI44L	20.9184	3.9537	2.4035	6.67E−54	1.75E−50	Up
IFITM1	70.1519	15.0070	2.2248	7.26E−11	1.14E−08	Up
C15orf38-AP3S2	1.6975	37.8404	−4.4784	2.33E−91	1.83E−87	Down
MYL9	3.6805	32.9425	−3.1620	7.84E−46	1.12E−42	Down
SEMA5A	1.0712	8.5887	−3.0032	3.11E−51	6.97E−48	Down
INHBE	0.9507	7.1699	−2.9149	9.39E−15	2.08E−12	Down
MED29	12.8370	67.4941	−2.3945	0.000982147	0.027948723	Down
IGFBP3	16.3876	84.3768	−2.3642	1.47E−61	5.76E−58	Down
ACTA2	15.4877	67.7854	−2.1298	8.80E−15	1.98E−12	Down
SNHG18	1.8375	6.8503	−1.8984	9.99E−07	8.48E−05	Down
SIRPA	2.3110	8.5946	−1.8949	7.37E−20	2.63E−17	Down
TGFB1I1	2.3757	8.0186	−1.7550	6.96E−10	9.76E−08	Down
EDN1	3.2891	10.6931	−1.7009	8.88E−07	7.58E−05	Down
CD24	20.8444	65.1091	−1.6432	3.87E−19	1.27E−16	Down
ZNF48	2.2354	6.9427	−1.6350	9.70E−06	0.000622028	Down

Further, the Database for Annotation, Visualization, and Integrated Discovery (DAVID) was introduced for GO and KEGG-enrichment analysis to study the biological function of DEGs. DEGs are mainly rich in the multi-organism process, immune system process, response to stimulus, negative regulation of the biological process, signaling etc. (Fig. 5f and Table S1). In cellular component terms, the DEGs were mainly involved in the organelles, extracellular region, extracellular region part, membrane-enclosed lumen, and cell part. For molecular functions, the DEGs were mainly enriched in binding receptor regulator activity, molecular function regulator, protein tag, and enzyme regulator activity. Through KEGG pathway analysis of the DEGs, we could find that DEGs is mainly enriched in cell growth and death, cell motility cellular community, transport, and catabolism in cellular processes, and is mainly enriched in environment information processing, signal transduction, signaling molecules, and interaction in membrane transport (Fig. 5g and Table S2). To explore the functional enrichment of the identified DEGs, we submitted the upregulated genes and downregulated genes to the DAVID for functional pathway analysis including GO (Fig. 5h and Table S3). The most significantly altered pathway was a response to the virus, a response to type I IFN, a negative regulation of viral life cycle, an innate immune response, etc.

Finally, we downloaded these differential genes through the TCGA database and analyzed the linear correlation with the seq-HTSeq-FPKM-UQ value of FAM64A in PCa patients, and found that the expression levels of SNHG18, IFIT3, OAS1, PMSB9, OAS2, UBE2L6, IGFBP3, ZNF48, and the expression level of FAM64A are linearly correlated and consistent with the sequencing results (Fig. 5i). The dimerization of the IFN receptor initiates the phosphorylation of JAK1, which phosphorylates and activates STAT1 and STAT2 proteins. These proteins form a well-characterized complex IFN-stimulated gene factor 3 (ISGF3) with interferon regulatory factor 9 (IRF9). ISGF3 translocates to the nucleus and binds to IFN-stimulated response elements (ISREs) in the gene promoter, resulting in IFN-stimulated gene transcription [11]. This is a classic pathway related to type I IFN immunity signaling pathway. FAM64A expression was strongly associated with the expression genes of immunity and type I interferon by JAK–STAT-signaling pathway. Compared with the control group, the phosphorylation levels of STAT1 and STAT2 in LNCaP and 22Rv1 cells silenced by FAM64A were reduced, whereas the overall increase in the protein levels of IRF9 (Fig. 5j). Taken together, these data confirm the concept that FAM64A inhibition modulates the expression of IFN and immunologically relevant transcripts by the JAK–STAT-signaling pathway and highlights the complexity of this regulation.

## Discussion

The present study is the first to identify FAM64A as an oncogenic factor in process of PCa. In this study, we combine PCa cells, human PCa samples, and bioinformatics analysis to explore the expression, regulation, function, and mechanism of FAM64A in PCa through the DHT/AR axis. FAM64A modulates the expression of IFN and immunologically relevant transcripts by the JAK–STAT-signaling pathway.

We found that the expression of FAM64A in PCa cell and tissue samples was higher than that in normal ones, and was related to the patient’s T stage, Gleason score, lymph node metastasis, number of positive lymph nodes, overall survival, and disease-free survival by analysis of the TCGA database. In addition, we found that FAM64A promoted not only the proliferation and migration/invasion of PCa cells but also the cell cycle, which suggests that FAM64A may be closely related to the occurrence and progression of PCa. Several studies have shown that overexpression of FAM64A accelerated the proliferation, migration, and invasion of esophageal cancer cells, breast cancer, pancreatic cancer, lymphoma, and other tumor tissues than normal tissues [7–9, 12]. Arcangelo et al. reported that FAM64A plays a role in controlling cell proliferation and can be regarded as a sign of proliferation [13]. In glioblastoma cells, when the cells enter the S phase, FAM64A would be progressively up-regulated as the cell cycle progresses, and the FAM64A biological protein level was the highest in the G2 phase [13]. This finding reinforces the idea that FAM64A promotes PCa cell proliferation by promoting the G2 to M phase transition.

As a nuclear transcription factor and a steroid hormone receptor, AR plays an important role in the growth of PCa [14–16]. In canonical AR signaling, binding of androgens to AR causes the dimerization and activation of AR. AR dimer translocating into the nucleus binds to the promoters/enhancers of target genes and recruits RNA polymerase-II to initiate gene transcription [17]. In this study, it is showed that AR expression in PCa tissues is positively correlated with FAM64A. And the expression of AR and FAM64A are increased gradually following the increasing concentration of DHT. In addition, Chip-qPCR and luciferase reporter analysis verified that AR can bind to FAM64A promoter and promote its transcription. These results indicate that FAM64A is a new target gene of AR in PCa. In line with previous reports, we found that DHT treatment significantly accelerated cell proliferation, cell migration/invasion, and cell cycle progression. However, this effect was markedly diminished by knocking down endogenous FAM64A in LNCaP and 22Rv1 cells. Consistently knockdown of FAM64A by its siRNA impaired the DHT/AR-enhanced expression of cyclin D and cyclin B1 in LNCaP and 22Rv1 cells.

Advanced PCa relies on the internal and external mechanisms of a variety of cancer cells to promote tumorigenesis, evade immune surveillance and restrain IFN signaling, and actively block immune responses [18, 19]. Whole-genome sequencing of androgen-binding sites in PCa cell lines also demonstrated that GO functional enrichment analysis includes death, immune system process, multi-biological process, and virus reproduction [20, 21]. Using transcriptome sequencing, IGFBP3, CCL5, TGFb, and CD24 are related to immune surveillance, SNHG18, IFIT3, OAS1, OAS2, and UBE2L6 are related to IFN signaling. SNHG18 inhibits the nuclear and cytoplasmic transport of ENO1 to promote the occurrence and metastasis of glioma [22]. IFIT3, a member of IFN-induced protein with tetratricopeptide repeats gene family mediating a variety of protein–protein and protein–RNA interactions, is involved in various cellular regulations [23]. IFITs are reported as prognostic markers to determine the clinical outcome of many cancers, such as glioblastoma, hepatocellular carcinoma, breast cancer, and pancreatic cancer [24–27]. OAS1 and OAS2 are composed of IFN-induced antiviral enzymes, which lead to the instability of virus-derived dsRNA with RNase L function, and the enzyme function has been well characterized [28]. Ubiquitin-conjugating enzyme E2 L6 (UBE2L6), an IFN-inducible ubiquitin E2, modulates ISGylation in leukemic cells to strongly interfere with the neutrophil differentiation of ATRA-treated APL cells [29]. Studies have shown that CD4+ T cells may activate STAT3 through CCL5 to affect the chemosensitivity of PCa, and the expression of MHC I increases the immunogenicity of PCa tumor cells [30]. Nuclear IGFBP3 is bound to chromatin and regulates the expression of chemokines and cytokines (including CCL5 and TGFb). These factors contribute to the establishment of an immunosuppressive tumor environment by driving the increased number of regulatory T cells in tumors [31]. CD24 was specifically overexpressed in the tumor compartment, orchestrates a novel innate immune checkpoint through interaction with the inhibitory receptor sialic acid-binding Ig-like lectin 10 (Siglec-10) on tumor-associated macrophages in primary ovarian and breast cancer samples [32]. The JAK–STAT pathway has been identified as the key node of many pathways, and mediates a large number of processes required for the occurrence and development of tumor cells. It determines the fate of T lymphocytes, and regulates their recruitment, survival, status, and eventual death [33, 34]. IL17 can promote the invasion of PCa which is secreted by T cells by increasing several EMT transcription factors and MMP7. However, the collaboration between type I and type II IFN supports innate and adaptive anti-tumor immune responses, STAT1 protein plays an important role in the interconnection of these pathways [35]. Under the stimulation of type I IFN (such as IFN α and IFN β), transcription factors such as signal converters and transcription activators (such as STAT1 and STAT2) are phosphorylated, and together with IRF9, the ISGF3 trimeric complex is formed. The complex localizes to the nucleus, and its response elements (ISREs) bind to DNA and drive the expression of IFN-stimulating genes (ISGs) [11]. In melanoma and lung cancer models, impaired phosphorylation of STAT1 in response to IFN-γ results in low MHC induction rate and reduced IFN-γ sensitivity due to defective JAK signal transduction [36]. Activation of STAT has been implicated in treatment naïve and advanced PCa, the levels of activated, nuclear STAT proteins were significantly elevated in metastatic CRPCa compared with benign prostatic hyperplasia (BPH) [37]. At the same time, JAK/STAT and AR pathways have synergistic functions in tumor cells, which may be related to the progression of PC [38, 39]. We have also proved that the expression of FAM64A can affect the immunity of PCa cell lines and the expression of IFN-relevant transcripts through the JAK–STAT pathway. Therefore, the activation of AR by DHT leads to the high expression of FAM64A which promotes the proliferation and invasion of PCa cell lines, FAM64A could impact the expression of immunity and IFN relevant transcripts by the JAK–STAT pathway in PCa.

## Conclusion

In summary, FAM64A was up-regulated in both PCa tissues and cell lines, and the overexpression of FAM64A in PCa cells could promote cell proliferation and invasion, and induce cell cycle progression. Moreover, AR activated by DHT bind the promoter sequence of the FAM64A gene to transcriptionally activate FAM64A and further accelerated the function of FAM64A in PCa. In addition, the lever expression of FAM64A might participate in the process of PCa by regulating the expression of immune and IFN genes by the JAK–STAT pathway in PCa. Understanding the mechanism of FAM64A might provide us a new therapeutic target for PCa treatments.

## Materials and methods

### Cell culture and database mining

Human prostatic epithelial cell line (P69) and PCa cell lines (PC-3, DU145, LNCaP, and 22Rv1) were purchased from Prof. Jun Wan, Shenzhen PKU-HKUST Medical Center, Shenzhen, China and cultured in RPMI-1640 medium supplemented with 10% fetal bovine serum (FBS, Gibco, Waltham, MA). Cells were maintained at 37 °C with 5% CO_2_. For all experiments involving dihydrotestosterone (catalog number: MB5484, Melonepharma, Dalian, China,) treatments, the medium was replaced with a phenol red-free medium containing 10% charcoal-stripped FBS.

The TCGA data was downloaded to investigate the expression of gene mRNA levels in PCa and normal prostate tissues, and the correlation of FAM64A expression and clinicopathological characteristics of patients with PCa.

### Transfection and RNA silencing

PCa cells were transfected with siRNAs using Lipofectamine 3000 (Invitrogen Inc., CA, USA). FAM64A-specific siRNAs (siR-FAM64A), AR siRNAs (siR-AR), or the corresponding negative control siRNAs (siR-NC) were synthesized by GenePharma Co., Ltd (Suzhou, China). The siRNAs’ sequences were as followed (actually, we have two pairs of siR-AR and siR-FAM64A, and chose one pair of them, Materials S1, Fig. S1):

siR-AR forward: 5′-GAAAAUGAUUGCACUAUUGATT-3′,

siR-AR reverse: 5′-UCAAUAGUGCAAUCAUUUCTT-3′;

siR-FAM64A forward: 5′-GGCUCAUGCCCACCCAUTT-3′,

siR-FAM64A reverse: 5′-AUGGGUGGGCAUGUGAGCCTT-3′;

siR-NC forward: 5′-UUCUCCGAACGUGUCACGUTT-3′,

siR-NC reverse: 5′-ACGUGACACGUUCGGAGAATT-3′.

### Western blotting

Total protein was extracted from the PCa cell pellets with RIPA Lysis Buffer (Beyotime biotechnology, Shanghai, China). Protein samples were loaded into 10% SDS–PAGE and subjected to electrophoretic analysis and subsequent blocking. Membranes were incubated with the primary antibodies (overnight at 4 °C) and the relevant secondary antibodies (1 h at room temperature). The antibodies were used in this study as follows: anti-AR (Santa Cruz biotechnology, Inc., USA, 1:1000), anti-FAM64A (Thermo Fisher Scientific, Inc., USA, 1:1000), anti-cyclin B1 (Cell Signaling Technology, Inc., MA, USA, 1:1000), anti-β-tubulin (Abcam, UK, 1:5000), anti-stat1 (Cell Signaling Technology, Inc., MA, USA, 1:1000), anti-phospho-stat1 (Cell Signaling Technology, Inc., MA, USA, 1:1000), anti-stat2 (Cell Signaling Technology, Inc., MA, USA, 1:1000), anti-phospho-stat2 (Cell Signaling Technology, Inc., MA, USA, 1:1000), anti-IRF-9 (Cell Signaling Technology, Inc., MA, USA, 1:1000), anti-rabbit secondary antibody (Cell Signaling Technology, Inc., MA, USA, 1:1000), anti-mouse secondary antibody (Cell Signaling Technology, Inc., MA, USA, 1:1000).

### RNA extraction and real-time qPCR

Total RNA was extracted from cells using the Trizol reagent (Invitrogen, Carlsbad, CA, USA) and 2 µg total RNA reverse-transcribed using the PrimeScript™ RT reagent Kit with gDNA Eraser (Takara, Tokyo, Japan). Real-time qPCR was performed with the SYBR Green MasterMix reagent (Takara, Tokyo, Japan) using the light cycler 480 II (Roche, USA). The human GAPDH gene served as endogenous control. The comparative 2^−△△Ct^ method was used to calculate gene expression levels. Each sample was analyzed in triplicate at least. The RNAs’ sequences were as follows:

GAPDH forward: 5′-CCACTCCTCCACCTTTGACG-3′,

GAPDH reverse: 5′-CTGGTGGTCCAGGGGTCTTA-3′;

FAM64A forward: 5′-CCTGAGAGTGTCTTGGGAG-3′,

FAM64A reverse: 5′-ACAGTGGGTGAGTGACTCTG-3′;

AR forward: 5′-AAGAGCCGCTGAAGGGAAAC-3′,

AR reverse: 5′-AGTTTCTTCAGCTTCCGGGC-3′.

### Immunohistochemistry staining for FAM64A

Immunohistochemistry staining was performed on Single spot tissue microarrays (TMA) slides (HProA150CS01, Shanghai Outdo Biotech Company) using the FAM64A rabbit antibody (1:20, Thermo Fisher Scientific, Inc. USA) and the rabbit streptavidin–biotin detection system (Beijing Zhongshan Golden Bridge Biotechnology Co., Ltd., Peking, China). The primary PCa specimens, which were excised from patients and confirmed including different Gleason scores from 6 to 10, were selected as PCa group; and the normal tissues next to the tumor were selected as an adjacent normal group. Of note, the result was assessed according to the intensity of staining (0, 1+, 2+, and 3+) and the percentage of positive cells (0 (0–10%), 1 (10–25%), 2 (26–50%), 3 (51–75%), and 4 (76–100%)) by two experienced pathologists. Finally, the product of the fraction of staining intensity and positive rate was used as the final score. Those less than or equal to the median value were considered as the low-expression group, while those higher than the median value were considered as the high-expression group.

### Chromatin immunoprecipitation (ChIP)

Following treatment, ChIP experiments with antibodies specific for AR (Santa Cruz biotechnology, Inc., USA) or normal rabbit IgG (Thermo Fisher Scientific, Inc., USA) were performed for three independent biological replicate experiments according to the manufacturer’s protocols of SimpleChIP^®^ Plis Sonication ChIP Kit 4C and RT Reagents (Cell signaling Technology, Inc., MA, USA) For ChIP-qPCR, DNA (ChIP-enriched and input) was subjected to quantitative PCR using primers specific to the sequences flanking the AR-binding elements within the FAM64A promoter.

### Dual Luciferase reporter assay

PCa cells were cotransfected with 50 pmol siRNAs and 30 ng a Renilla luciferase vector (pRL-TK) using Lipofectamine 3000 (Invitrogen Inc., CA, USA). Firefly and Renilla luciferase activities were determined at 48 h post-transfection with the Dual-Luciferase Reporter Assay System (Promega Corporation, Madison, USA) by a microplate luminometer (Tecan, Männedorf, Switzerland). The Renilla luciferase activity was used as the internal control to normalize the Firefly luciferase activity. All experiments were carried out in triplicate wells and repeated three times.

### Colony formation assay

1000 PCa cells per well were plated into six-well plates and cultured with the completed medium for 2 weeks. Colonies were washed twice with PBS and fixed with 4% paraformaldehyde for 15 min. Fixed colonies were stained with 0.05% crystal violet for 20 min; and then the number of colonies was counted and photographed under an inverted microscope (Nikon, Tokyo, Japan).

### Wound healing assay

PCa cells were suspended and seeded into a six-well plate. After the cells attached to the bottom and formed a monolayer, linear wounds were created by scratching the center of the monolayer with a 10 μl pipette tip. The detached cells were carefully washed off with PBS thrice. Then, 2 mL of culture medium without FBS was added to each well, and the wounds were photographed at 0, 48, and 96 h under a microscope (Olympus, Japan). The width of each scratch wound was recorded and analyzed.

### Transwell invasion assay

Following transfection, PCa cells were seeded onto the basement membrane matrix (EC matrix, Chemicon, Temecula, CA) present in the insert of a 24-well culture plate. Complete culture medium was added to the lower chamber as a chemoattractant. After a further 48 h, the non-invading cells and EC matrix were gently removed with a cotton swab. Invasive cells located on the lower side of the chamber were fixed with 4% paraformaldehyde and stained with crystal violet, air-dried and photographed with a microscope (Nikon Elipse Ci, Japan).

### Cell cycle assay

PCa cells with siR-NC and siR-FAM64A cultured for 48 h were fixed overnight in 75% alcohol at 4 °C. The fixed cells were resuspended in PBS containing PI/RNase staining buffer (BD Biosciences, San Diego, CA), and incubated for 15 min at room temperature. Cells were subjected to flow cytometric analysis of DNA content using a flow cytometer (BD Accuri^TM^ C6 Plus, BD Bioscience, CA). The percentages of cell cycle distribution were calculated by Flowjo VX software.

### Transcriptome sequencing and data processing

Total RNA was extracted using the Total RNA Extractor (Trizol) kit (B511311, Sangon, China) according to the manufacturer’s protocol, and treated with RNase-free DNase I to remove genomic DNA contamination. RNA integrity was evaluated with a 1.0% agarose gel. Thereafter, the quality and quantity of RNA were assessed using a NanoPhotometer^®^ spectrophotometer (IMPLEN, CA, USA) and an Agilent 2100 Bioanalyzer (Agilent Technologies, CA, USA). The high-quality RNA samples were subsequently submitted to the Sangon Biotech (Shanghai) Co., Ltd. for library preparation and sequencing.

### Statistical analysis

All error bars in graphical data represent mean ± SD. Pearson correlation coefficients were used to determine the correlation between the expressions of two genes. Correlation analysis between FAM64A expression and various clinical-pathological variables was performed by *χ*^2^ test and Fisher’s exact test. All statistical analysis was performed using SPASS 19.0 and GraphPad Prism 6 software and *P* < 0.05 was considered as a statistically significant difference between groups.

## Supplementary information

Supplement

## Data Availability

The dataset used during this study is available from TCGA and NURSA database.
