# Supplementary material for: FAM64A is an androgen receptor-regulated feedback tumor promoter in prostate cancer
Source: Cell Death Dis. 2021 Jul 2;12(7):668. doi: 10.1038/s41419-021-03933-z (PMC8253826; doi:10.1038/s41419-021-03933-z)

| **Table S1. Top5 enriched GO terms of DEGs** | | | | | |
| --- | --- | --- | --- | --- | --- |
| Ontology | Term | GO.ID | Significant | Annotated | Qvalue |
| biological process | multi-organism process | GO:0051704 | 43 | 2661 | 8.74E-08 |
| biological process | immune system process | GO:0002376 | 46 | 3502 | 9.28E-06 |
| biological process | response to stimulus | GO:0050896 | 82 | 9715 | 0.000552429 |
| biological process | negative regulation of biological process | GO:0048519 | 54 | 5294 | 0.001306187 |
| biological process | signaling | GO:0023052 | 64 | 7054 | 0.00402168 |

| **Table S2. GO Enrichment  terms of function** | | | | | |
| --- | --- | --- | --- | --- | --- |
| GO.ID | Term | Ontology | Significant | Annotated | Pvalue |
| GO:0009615 | response to virus | biological process | 29/112 | 318/18974 | 1.40E-26 |
| GO:0060337 | type I interferon signaling pathway | biological process | 16/112 | 121/18974 | 1.50E-17 |
| GO:0034340 | response to type I interferon | biological process | 16/112 | 125/18974 | 2.60E-17 |
| GO:0043207 | response to external biotic stimulus | biological process | 33/112 | 955/18974 | 4.70E-17 |
| GO:1903901 | negative regulation of viral life cycle | biological process | 13/112 | 104/18974 | 4.00E-14 |
| GO:0043901 | negative regulation of multi-organism process | biological process | 15/112 | 209/18974 | 1.60E-12 |
| GO:0045087 | innate immune response | biological process | 30/112 | 1175/18974 | 4.50E-12 |
| GO:0002252 | immune effector process | biological process | 32/112 | 1425/18974 | 2.20E-11 |
| GO:0019221 | cytokine-mediated signaling pathway | biological process | 23/112 | 726/18974 | 3.30E-11 |
| GO:0071345 | cellular response to cytokine stimulus | biological process | 24/112 | 895/18974 | 3.60E-10 |

**Table S3. Top5 enriched KEGG terms of DEGs**

| Group | Subgroup | Sign | Anno |
| --- | --- | --- | --- |
| Environmental Information Processing | Signal transduction | 17 | 1792 |
| Human Diseases | Cancers | 11 | 1087 |
| Organismal Systems | Immune system | 11 | 1259 |
| Human Diseases | Infectious diseases | 10 | 1281 |
| Cellular Processes | Cellular community | 5 | 545 |

Materials S1

siR-AR 1, forward: 5′-GAAAAUGAUUGCACUAUUGATT-3′,

reverse: 5′-UCAAUAGUGCAAUCAUUUCTT-3′;

siR-AR 2, forward: 5′-GAAGGGAGGUUACACCAAATT-3′,

reverse: 5′-UUUGGUGUAACCUCCCUUGTT-3′；

siR-FAM64A 1, forward: 5′-GGCUCAUGCCCACCCAUTT-3′,

reverse: 5′-AUGGGUGGGCAUGUGAGCCTT-3′;

siR-FAM64A 2, forward: 5′-GCUUCUCGGUGGCAGAACATT-3′,

reverse: 5′-UGUUCUGCCACCGAGAAGCTT-3′

Figure S1 The mRNA and protein level of AR and FAM64A in PCa cell lines LNCaP and 22Rv1 with infection of siRNA-AR and siRNA-FAM64A were noteworthy lower than that in the control group.


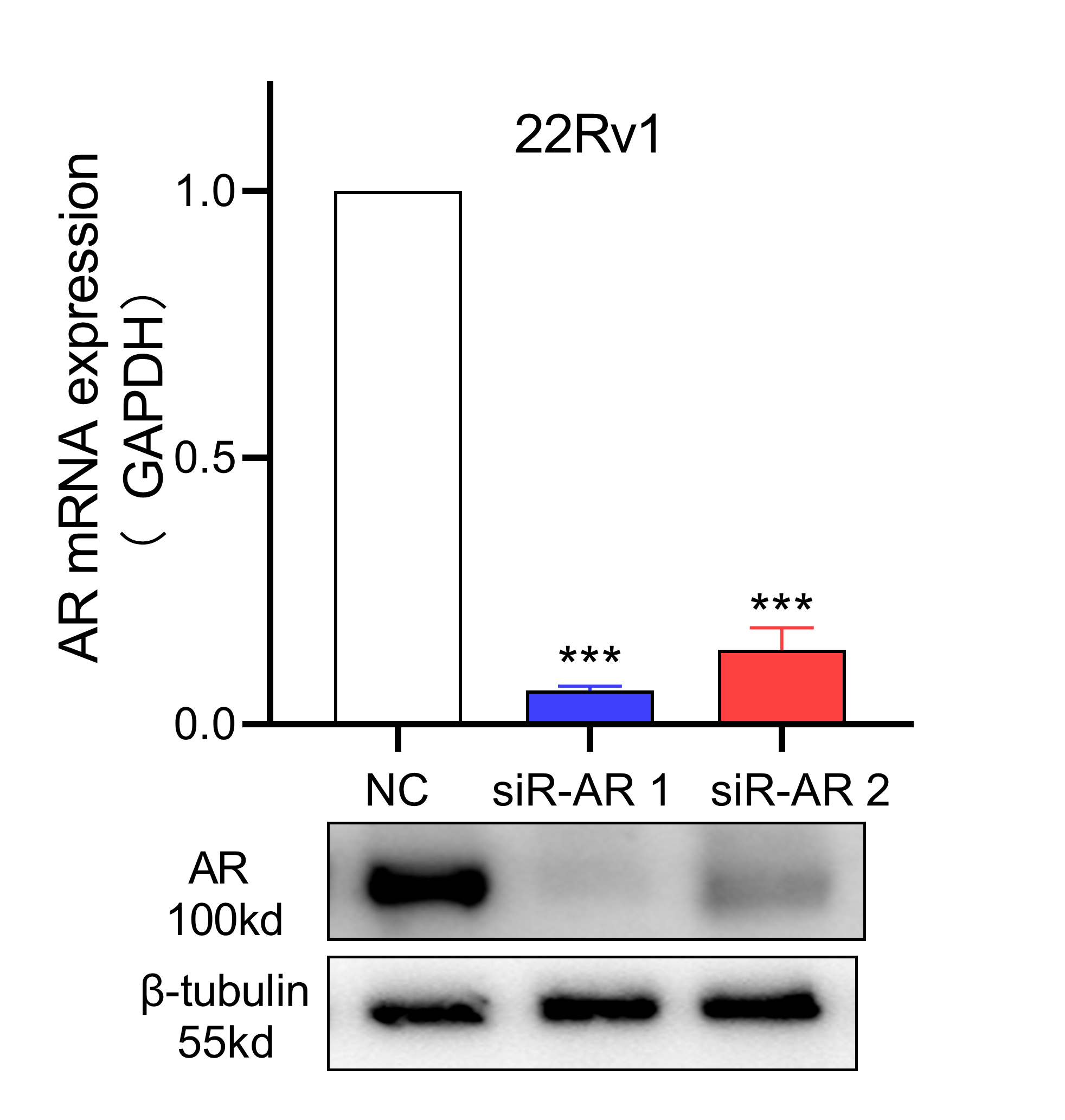

Supplement: Supplementary file 1 — Supplement [file 41419_2021_3933_MOESM1_ESM.doc]
